# Enhanced Drug Delivery for Cardiac Microvascular Obstruction with an Occlusion-Infusion-Catheter

**DOI:** 10.1007/s10439-023-03142-z

**Published:** 2023-01-21

**Authors:** Yannick Rösch, David Eggenberger, Yves Kuster, Lino Widmer, Sabrina Frey, Rob Schwartz, Cornelia Nef, Jens Ulmer, Dominik Obrist

**Affiliations:** 1 ARTORG Center for Biomedical Engineering Research, University of Bern, Freiburgstrasse 3, 3010 Bern, Switzerland; 2 Institute for Microtechnology and Photonics, OST University of Applied Sciences, Buchs SG, Switzerland; 3 CorFlow Therapeutics AG, Baar, Switzerland; 4 matriq AG, St. Gallen, Switzerland

**Keywords:** MVO, Intracoronary drug infusion, Balloon occlusion, Controlled flow infusion, STEMI, Coronary circulation, Microfluidic chip, Left-heart mock loop

## Abstract

Microvascular Obstruction (MVO) is a common consequence of acute myocardial infarction. MVO is underdiagnosed and treatment is often nonspecific and ineffective. A multi-scale *in-vitro* benchtop model was established to investigate drug perfusion in MVO affected microcirculation. The central element of the benchtop model was a fluidic microchip containing channels with diameters between $555$ and 50 *μ*m representing $2\%$ of the microvascular tree fed by the left anterior descending artery (LAD). The outlets of the chip could be closed to mimic MVO. Two methods for intracoronary infusion of pharmacologic agents (simulated by dye) to regions with MVO were investigated using an occlusion-infusion catheter. The first case was a simple, bolus-like infusion into the LAD, whereas the second case consisted of infusion with concomitant proximal occlusion of the LAD phantom with a balloon. Results show that local dye concentration maxima in the chip with MVO were 2.2–3.2 times higher for the case with proximal balloon occlusion than for the conventional infusion method. The cumulated dose could be raised by a factor 4.6–5.2. These results suggest that drug infusion by catheter is more effective if the blood supply to the treated vascular bed is temporarily blocked by a balloon catheter.

**Supplementary Information:**

The online version contains supplementary material available at 10.1007/s10439-023-03142-z.

## Introduction

Up to 40–60% of all ST-elevation myocardial infarction (STEMI) patients suffer from microvascular obstruction (MVO) following successful recanalization of blocked epicardial coronary arteries with balloon and stent.^4,8,17^ MVO may be caused by residual particles of the primary thrombus (microthrombi), tissue swelling or collapsed vessels in the microcirculation of the myocardium.^1,9,16,21,23^ These poorly perfused or even non-perfused regions of the tissue are often not detected in the catheter laboratory due to the limited resolution of fluoroscopic angiography^5^ and have substantial negative prognostic impact.^4,6,17^ The gold standard for MVO diagnosis is non-perfusion within regions of Late-Gadolinium-Enhanced Cardiac Magnetic Resonance Imaging (LGE-CMRI).^3^ MRI however is impractical in acute diagnosis and treatment of STEMI patients.

Even when MVO is diagnosed, treatment strategies are not well established. Direct microvascular access *via* catheter is impossible because the affected vessels are too extensive and too small.^21^ The efficacy of systemic administration of thrombolytic drugs is questionable and direct intracoronary infusion of the drugs show no significant advantage over intravenous infusion.^2,10,15,19^

**Figure 1:**
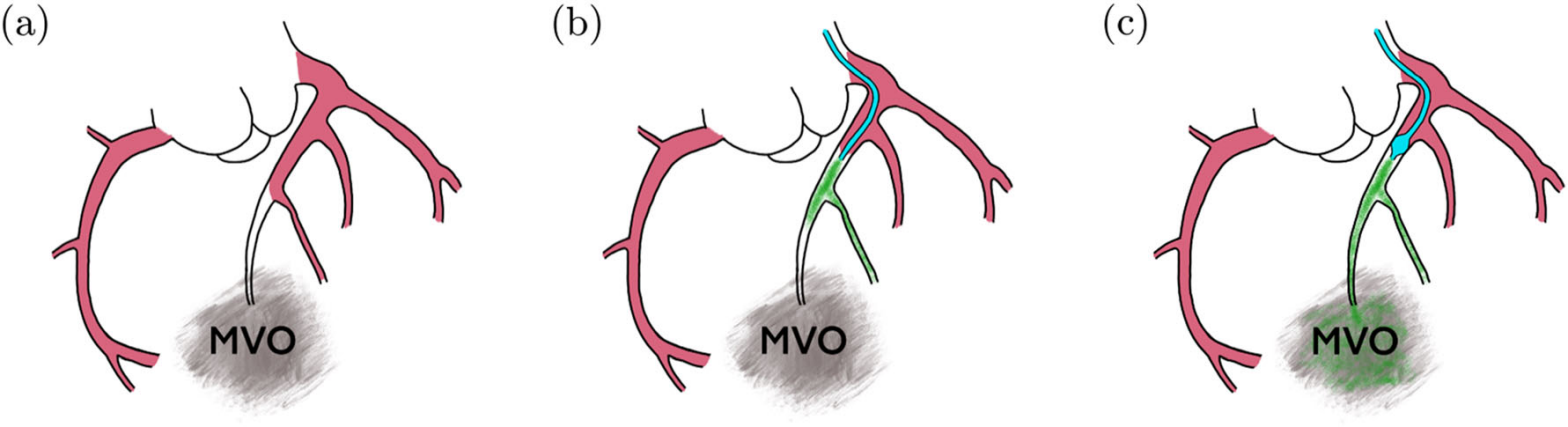
(a) Schematic of an MVO region with poor perfusion (b) Treatment of MVO with intracoronary infusion. Most of the drug is washed out through non-occluded vessels. (c) Treatment of MVO with balloon-occlusion infusion leading to enhanced drug delivery toward the MVO.

The limited success of pharmaceutical treatment may be directly related to the poor perfusion of the affected tissue. It is conceivable that the local blockage of blood flow hinders the drug from reaching the MVO lesion and that most of the drug is flushed away through the healthy part of the coronary microcirculation (Figs. 1a, 1b).

This study addresses the problem of drug delivery to MVO and explores a catheter-based method to enhance the efficacy of drug administration. It exploits a novel technology which has been recently proposed for the quantitative diagnosis of MVO.^24^ This technology uses a multi-lumen occlusion-infusion-catheter to temporarily occlude with a balloon the affected coronary artery at the site of the primary myocardial infarction. Distal infusion of buffered isotonic solution trough a second lumen of the catheter yields a controlled perfusion of the distal vascular tree at a known infusion flow rate $Q$. Simultaneous measurement of the pressure $p$ with a pressure wire in the third catheter lumen yields information about the hydraulic resistance, $R = p/Q$, of the vascular bed distal to the occluding balloon. It has been shown that this method can detect the increased vascular resistance due to MVO in pig models.^22,24^

The main objective of this study is to find out if this occlusion-infusion catheter could be used to administer the drug while the balloon is inflated and if this yields a higher efficacy of drug delivery than an intracoronary drug administration by a standard catheter. To this end, the multi-scale coronary circulation model from Thirugnanasambandam *et al*.^24^ has been further developed and now comprises a microfluidic chip that models a small partition of the myocardial microcirculation. This transparent chip allows for a quantitative assessment of the drug transport in the microchannels of the chip. It will be shown that an infusion with proximal balloon occlusion leads to significantly higher drug concentrations and longer drug dwelling times in the affected regions.

## Materials and Methods

### Occlusion-Infusion Catheter

**Figure 2:**
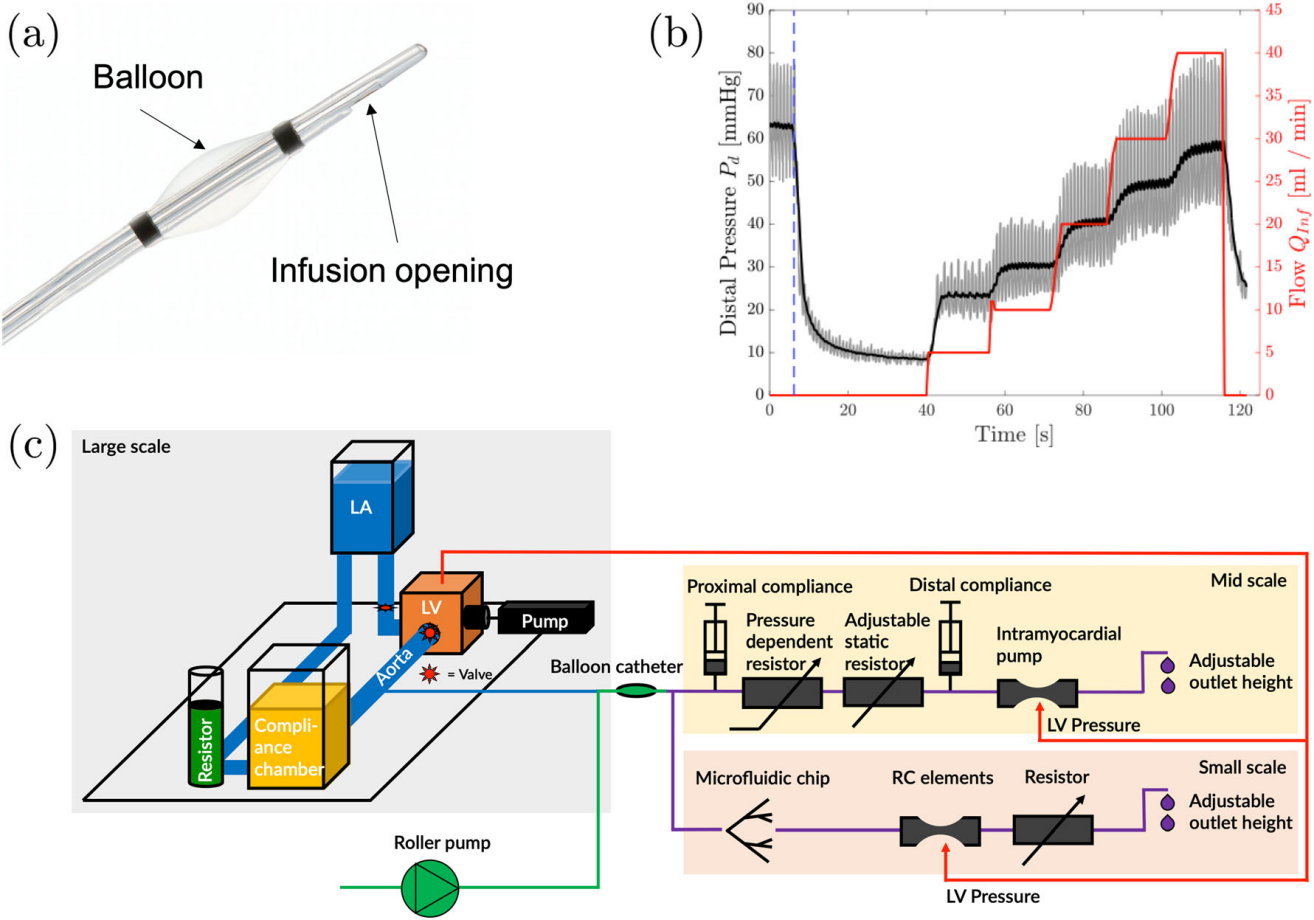
(a) Occlusion-infusion catheter with a balloon to block a coronary artery proximally, a lumen for controlled fluid infusion and a pressure wire for distal pressure measurement. (b) Distal coronary pressure (grey) with mean pressure (black) during a diagnostic occlusion-infusion sequence OIS ($Q_{{{\text{Inf}}}}$ shown in red, timepoint of balloon inflation marked with blue dashed line) performed on a pig with MVO. (c) Schematic of the left-heart mock loop and the coronary circulation model with impedance elements and microchip.

For the controlled infusion into the coronary circulation the Controlled Flow Infusion (CoFI) system (CorFlow Therapeutics AG, Baar, Switzerland) is used. The central part of this system is a custom multi-lumen rapid-exchange occlusion-infusion balloon catheter (Fig. 2a). This catheter can occlude an epicardial blood vessel by inflating a balloon. Distal to the balloon a pressure sensing guidewire (OptoWire Deux, OpSens, Québec, Canada) is used to measure the distal blood pressure. The third lumen is used to simultaneously infuse fluids distal to the balloon. The infusion is driven by a roller pump (LiveTec LiveCool Infusion Pump, livetec Ingenieurbüro GmbH, Lörrach, Germany).

In the diagnostic application, this catheter is used to perform an occlusion-infusion sequence (OIS) where buffered isotonic fluid is infused with stepwise increasing flow rates ($Q = 5, 10, 20, 30, 40\, {\text{mL/min}}$) while the distal pressure $p_{d}$ is measured. OIS results from a pig with MVO (Fig. 2b) are used for tuning the coronary circulation model (see “Coronary Circulation Model” section) such that $p_{d}$ measured during the OIS in the coronary model matches the OIS results from the pig. Details on the procedure for model tuning are given in Reference 24.

### Coronary Circulation Model

The coronary circulation model was designed as a multi-scale benchtop model comprising three components: a left-heart mock loop (large scale), a coronary model (mid scale) and a microfluidic chip (small scale). The experimental setup with left-heart mock loop and mid-scale coronary model was already used in a previous study (described in detail in Reference 24). Here, we provide only an overview of these two components and focus on the microfluidic chip which was added to the setup to model the flow characteristics in the myocardial microcirculation with and without MVO.

The validated left-heart mock loop from Jahren *et al*.,^11^ driven by a computer-controlled custom-built piston pump, was used to generate physiological pressure-flow-relations in an aortic root phantom. The pump consisted of a stepper motor (34S42, Jenaer Antriebstechnik, Jena, Germany) and a ball screw (ELK40, Bahr Modultechnik GmbH, Luhden, Germany), connected with the piston. The action of the stepper motor was precisely controlled with a control unit (XEMO R, Systec GmbH, Münster, Germany) which allowed to operate the piston with a specific, user-defined velocity profile. One of the two ostia of the aortic root was connected to the coronary model which comprised several hydraulic impedance elements (Fig. 2c) to model the dynamics of coronary blood flow. Details on the impedance elements and their effect on the flow through the coronary model are given in Reference 24, where it is also shown that this model reproduces a classical coronary waveform with peak coronary flow during diastole. The model was validated against results from OIS in a pig model.^24^

In addition to the model described,^24^ a parallel branch with a microfluidic chip modelling the arteriolar tree (hereafter called microchip) was added to the coronary model.

**Figure 3:**
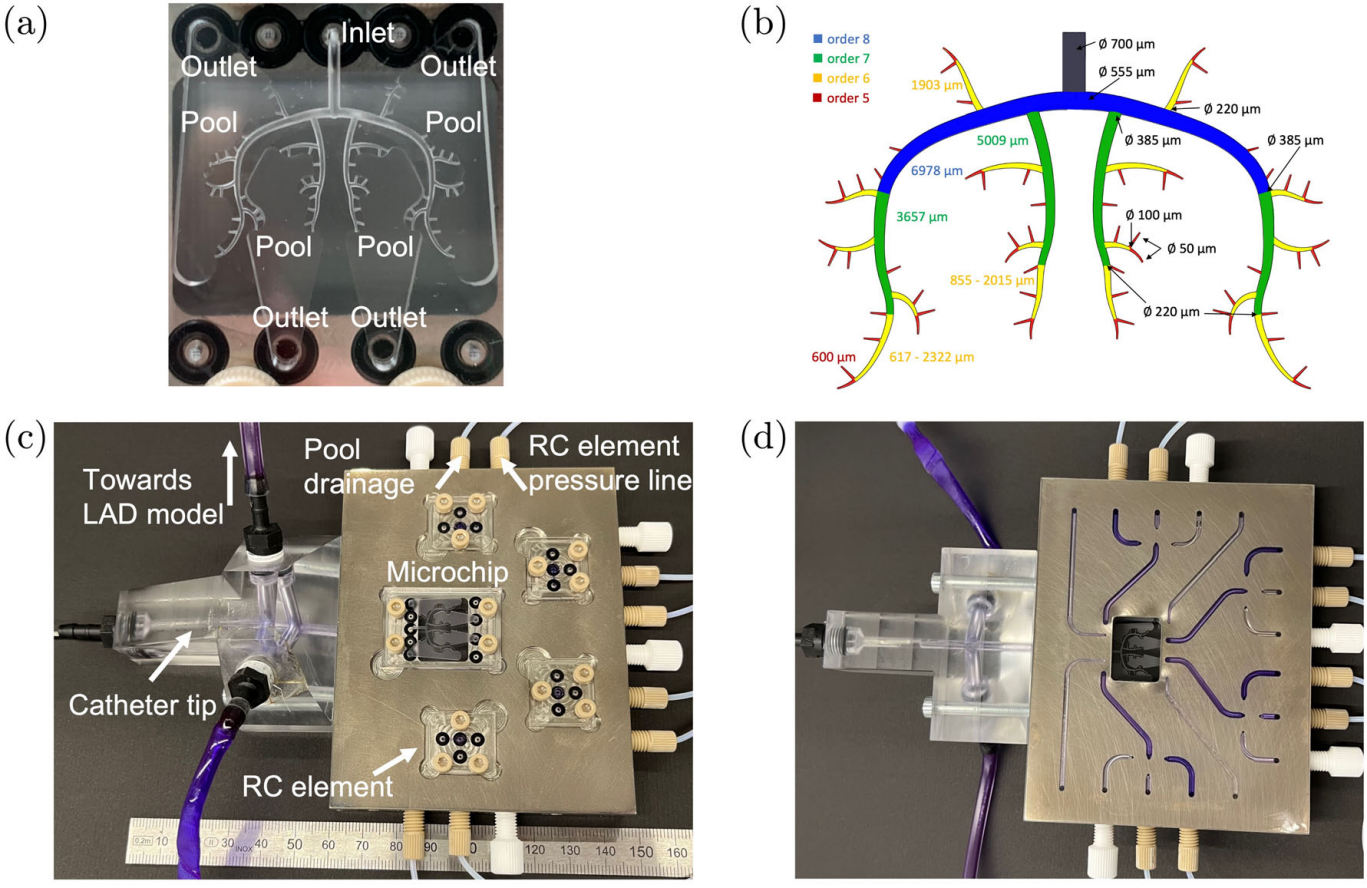
(a) Top view of the microfluidic chip. (b) Schematic of the microchip design with segment lengths (left side) and segment diameters (right side). (c) Top view of microfluidic backplate with microchip and RC elements (d) Bottom view of the microfluidic backplate with microchip and RC elements (d) Bottom view of microfluidic backplate with microchip and RC elements.

This microchip was designed to model the anatomy of the myocardial microcirculation based on anatomical data given in Kassab *et al*.^13^ and Kalsho *et al*.^12^ It was integrated in the coronary circulation model as shown in Fig. 2c. The microchip modelled the arteriolar tree with four generations of vessels ranging from diameters of $555\, \mu {\text{m}}$ (order 8) down to $50\, \mu {\text{m}}$ (order 5), as shown in Fig. 3. The represented part of the arteriolar tree corresponds approximately to $2 \%$ of the microvascular tree fed by the left anterior descending coronary artery (LAD). The range of diameters was chosen because previous studies found microthrombi causing MVO predominantly in vessels with diameters between $120$ and $40\, \mu {\text{m}}$.^21^ Smaller vessels of the microcirculation were not explicitly represented in the microchip. Instead, the outlets of the smallest vessels on the microchip were connected to four separate pools. The outlets of these pools were connected to hydraulic impedance elements to model microvascular compliance and flow dependent resistance. These flow and compliance tuners^18^ (RC elements) were built out of two PMMA substrates with embedded micro-milled channels. The channels from the two substrates overlapped to form a chamber which was separated in the middle by a thin PDMS foil $(20\,\mu {\text{m}}$ thickness) (Elastosil, Wacker Chemie AG, Munich, DE). This foil was bonded to the PMMA substrates using 3-Aminopropyltriethoxysilan (3-APTES) as linker.^14,25^ While the lower channel was directly connected the outflow of a microchip pool, the upper channel was connected by a tube to the left ventricle (LV) of the left-heart mock loop. High LV-pressure during systole lead to a deflection of PDMS foil which obstructed the lower channel. During diastole, LV pressure decreased while the pressure in the lower channel increased, such that the lower channel reopened. This introduced the myocardial pumping effect due to ventricular pressure and added at the same time compliance compensation for the rigid channels of the microchip.

In addition, an adjustable resistance element was added distal to the microchip to model the hydraulic resistance of the distal microvascular bed that was not included on the microchip. The single connections between the four separate outlets of the chip and the resistor could be fully clamped to induce MVO separately in the four vascular branches. Note that this resistance element and the clamps were distal to the RC elements such that there remained some vascular compliance even when the resistance element fully blocked the flow in a specific branch.

To comply with the vascular volume represented in the microchip, only a small fraction of $2 \%$ of the fluid in the coronary model was passed through this branch of the coronary model.

The microfluidic chip was fabricated by a laser-assisted etching process. Fused silica glass substrates (Siegert Wafer GmbH, Aachen, Germany) with dimensions of $50$ × $50$ × $3$ mm were structured with an ytterbium fiber laser (LightFab 3D Printer, LightFab GmbH, Aachen, Germany) at $1030\, {\text{nm}}$. After structuring, the laser-modified glass was selectively etched in $8\, {\text{M KOH}}$ (Sigma Aldrich AG, Buchs, Switzerland) at 80 °C until complete removal.^7^ The semi-circular channel structure etched at the glass surface where then covered with a 1 mm fused silica glass substrate (Siegert Wafer GmbH, Aachen, Germany) by using an 3-APTES PDMS bonding process.^14,25^ This method yielded semi-circular channels covered by a flat glass plane having comparable hydrodynamic properties to complete circular channels. Due to the etching process, the surface roughness was in the order of micrometers.

For better handling of the system, the microchip and the four RC elements were mounted on a titanium plate (microfluidic backplate) with milled channels and connectors to connect the different components. The volumes of these channels corresponded to vessels of which are proximal to the vessels represented in the microchip.

The microfluidic backplate featured a window such that a backlight (LED Microscopy backlight, $50\; {\text{mm}}, 9\, {\text{W}}$, BoliOptics, California) could be used to illuminate the microchip. A high-speed video camera (Body: Fastcam Mini AX 100, Photron; Lenses: $F 2.8/100\, {\text{mm}}$ Macro Lense, Samyang; Intermediate Ring set, Walimex) was mounted on the opposite side to capture the perfusion of the microchip with dye.

### Test Fluids

To run the left-heart mock loop and the coronary model, distilled water at room temperature was used. This is a simplification since the water representing blood has a lower dynamic viscosity ($\mu_{{{\text{H}}_{{2}} {\text{O}}}} = 0.001\, {\text{Pa s}}$) than blood ($\mu_{{{\text{Blood}}}} = 0.0035\, {\text{Pa s}}$).

Instead of a drug, a dye-solution self-diluted $0.1 \%$ crystal violet solution (Crystal Violet, $1 \%$ aqueous solution, Sigma Aldrich, diluted with distilled water to $0.1 \%$) was used. It was infused through the occlusion-infusion catheter into the LAD model (just before the branching of the lumped parameter model and the microchip).

### Experimental Protocol

First, the left-heart mock loop and the coronary model were tuned to match the flow-pressure curve (Fig. 2b) obtained from an OIS experiment in a pig (see Reference 24 for details on the matching procedure). Second, the flow through the microchip was tuned. To this end, the balloon of the catheter was inflated and $Q_{{{\text{Inf}}}}$ was set to $30\, {\text{mL/min}}$. The static resistor was then successively closed until the flow through the microchip was $0.6\, {\text{mL/min}}.$ Third, partial MVO was modelled by fully clamping the outlets of the two RC elements which were connected to the left side of the microchip. Fourth, the catheter was connected to a reservoir with the dye and was prefilled with the dye.

Two dye infusion protocols were compared. In the first case, called *no-balloon*, 15 mL dye were infused with the occlusion-infusion catheter over a period of 30 s. This case represented the control group. The second case, called *balloon*, started with the inflation of the balloon to occlude the vessel, followed by the infusion of 15 mL dye in 30 s (infusion phase), while keeping the balloon inflated. The balloon inflation was maintained for 60 s after the end of infusion (balloon-holding phase). After that, the balloon was deflated (total occlusion time $30 + 60\, {\text{s}}$).

The 15 mL infusion volume corresponds to one-third of the recommended dose for the treatment of a 90 kg heavy person with AGGRASTAT (Inf sol $12.5\, {\text{mg/}}250\, {\text{mL}}$), a (GP)-IIb/IIIa-receptor antagonist). This dosage is loosely motivated by the assumption that less drug is needed with the intracoronary application than in a systemic approach.

**Figure 4:**
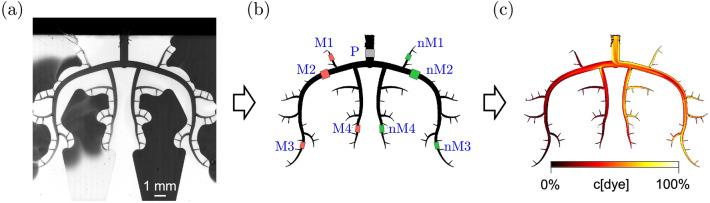
(a) Microchip partially perfused with dye (b) Network structure detected by post-processing algorithm. The rose, grey and mint rectangles show the regions of interest M1–M4 (MVO side), P (Parent vessel), nM1–nM4 (no-MVO side) (c) Heatmap; calculated dye concentration.

A camera recorded the illuminated microchip with a framerate of $10\, {\text{Hz}}$. The illumination and the camera shutter were configured to use the full illumination range of the camera (Fig. 4a). For all experiments the camera started recording 10 s before the start of the infusion and recorded 180 s in total.

Both cases (*balloon*, *no-balloon*) were tested with two different configurations of the coronary circulation model: all outlets of the microfluidic chip open (*no-MVO*) and with two outlets of the left side of the chip closed (*MVO*). For both configurations, the coronary model was matched to OIS data from the pig with MVO used in Reference 24. Aortic blood pressure was held at approximately $52/82\, {\text{mmHg}} \left( {6.9/10.9\, {\text{kPa}}} \right)$ and the heart rate was set to $50\, {\text{bpm}} \left( {0.83\, {\text{Hz}}} \right)$ (modelling a pig without MVO) and to $69\, {\text{bpm }}\left( {1.15\, {\text{Hz}}} \right)$ to model a pig with MVO.^24^ In total, 20 experimental runs for both infusion cases were performed with $50\, {\text{bpm}}$ (distributed over 4 days with new model tuning each day and 5 experiments per infusion case per day), and 10 experimental runs for both infusion cases with $69\, {\text{bpm}}$ (distributed over 2 days with new tuning each day and 5 experiments per infusion case per day).

### Data Analysis

For the evaluation of the recorded frames of the microchip (Fig. 4a), only the part containing the channels was extracted using a custom-made post-processing algorithm (Fig. 4b).

In a previous calibration experiment (see “Appendix”) a calibration curve was determined, which allowed to determine a time-resolved dye concentration for each pixel (Fig. 4c).

Distributed over the network, so-called Regions of Interest (ROI) were defined (Fig. 4b; the ROI are slightly enlarged for better visibility) which were distributed symmetrically on the *MVO* (M1, M2, M3, M4) and the *no-MVO* side (nM1, nM2, nM3, nM4) of the network. The ROI for the parent vessel was labelled P. The evaluated area in P was a square of length d which was placed 0.5d before the bifurcation (where d is the local vessel diameter). For M2 and nM2, the ROI was a rectangle of length 1.5d and width d which was placed in the middle of the order 8 vessel segment. All other ROIs were in order 6 vessels and had a length of 4d (width d), They were placed in the middle of the respective vessel segments following the first bifurcations to an order 5 vessel. For each ROI, the mean concentration over all included pixels was calculated. A moving average filter (width of a single heartbeat) was then applied to remove the pulsatility in the signal. This filtered signal was defined as the dye concentration $c\left( t \right)$ ranging from $0$ to $100 \%$.

Next to the concentration $c$, also the time a drug remains at a certain location may be important for the drug efficacy. Therefore, also the cumulated dose $D$ was calculated which was defined as the integral of the concentration ${\text{c}}$ over time,

$$D\left( t \right) = \mathop \smallint \limits_{0}^{t} c\left( {t^{\prime}} \right){\text{d}}t^{\prime}$$

For the purpose of the present study, we consider higher concentrations $c$ and higher cumulated doses $D$ to be beneficial for the drug delivery efficacy.

For the comparison of different experiments, the maximum concentration $c_{{{\text{max}}}}$ and the final cumulative drug dose $D_{{{\text{fin}}}}$ were defined as

$${\text{c}}_{{{\text{max}}}} = \mathop {\max }\limits_{t} c\left( t \right)$$

$$D_{{{\text{fin}}}} = D\left( {t = 180\;{\text{s}}} \right)\;$$

## Results

**Figure 5:**
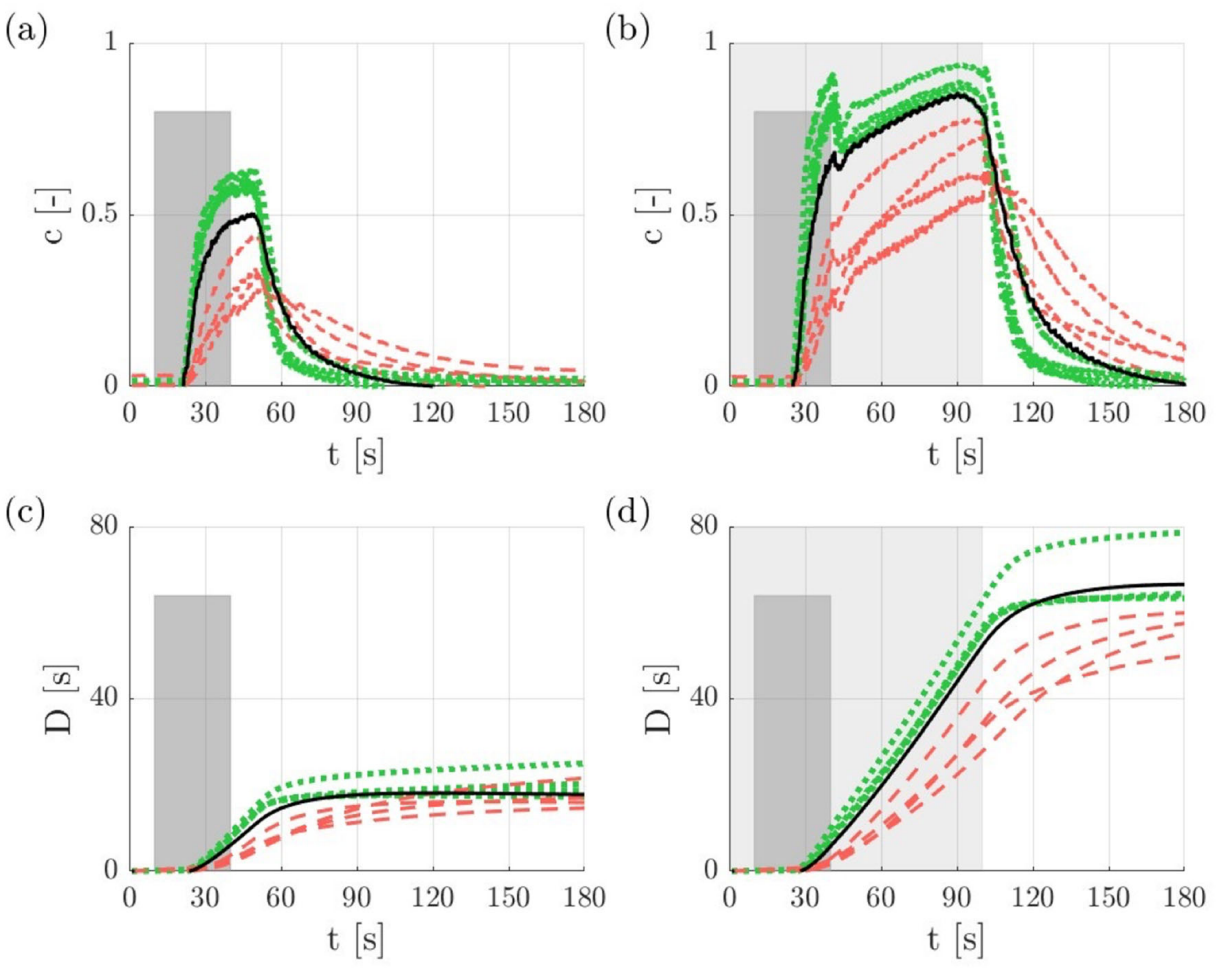
Concentration $c$ and cumulated dose $D$ for the parent vessel P (solid black line), no-MVO ROIs nM1-nM4 (dotted green lines), and MVO ROIs M1-M4 (dashed red lines) at $69\, {\text{bpm}}$: (a) & (c) no-balloon (dark grey: infusion phase), (b) & (d) balloon (dark grey: infusion phase, light grey: balloon-holding phase).

Figure 5 shows the evolution of $c$ and $D$ at different locations in the microchip during experiments with $69\, {\text{bpm}}$ (see also Supplementary video). In the *no-balloon* case (Fig. 5a) the concentrations rise after an initial lag of approximately 10 s, reach a maximum after end of the infusion period (dark grey area) and then decrease. The *MVO* side exhibits lower concentrations than the *no-MVO* side throughout the whole experiment. The concentration in the parent vessel lies between the *MVO* and *no-MVO* concentrations. In the *balloon* case (Fig. 5b) the concentrations also rise after an initial lag. At the end of the infusion phase, the concentrations on the *MVO* side are similar to the *no-balloon* case, while the *no-MVO* side reaches higher concentrations at the end of the infusion. Immediately after the end of the infusion phase, there are small dips in the concentration curves before the curves rise again for the remainder of the balloon-holding phase (light grey area) where the increase on the *MVO* side is higher than on the *no-MVO* side. The maximum concentrations $c_{{{\text{max}}}}$ on the *MVO* side in the *balloon* case are similar to $c_{max}$ on the *no-MVO* side in the *no-balloon* case. After balloon deflation the dye is washed similarly to the *no-balloon* case.

Figures 5c and 5d show the cumulated doses $D$ for the *no-balloon* case (c) and the *balloon* case (d).

For the *no-balloon* case, $D$ rises steeply during the infusion phase and continues growing until $c_{{{\text{max}}}}$ is reached. The following washout on the *no-MVO* side is faster than on the *MVO* side such that the cumulated doses on the no-MVO side plateau earlier than on the *no-MVO* side.

In the *balloon* case, the *no-MVO* side reaches higher $D_{{{\text{fin}}}}$ than on the *MVO* side. Nevertheless, all $D_{{{\text{fin}}}}$ (*MVO* and *no-MVO*) in the *balloon* case are higher than $D_{{{\text{fin}}}}$ in the *no-balloon* case.

**Figure 6:**
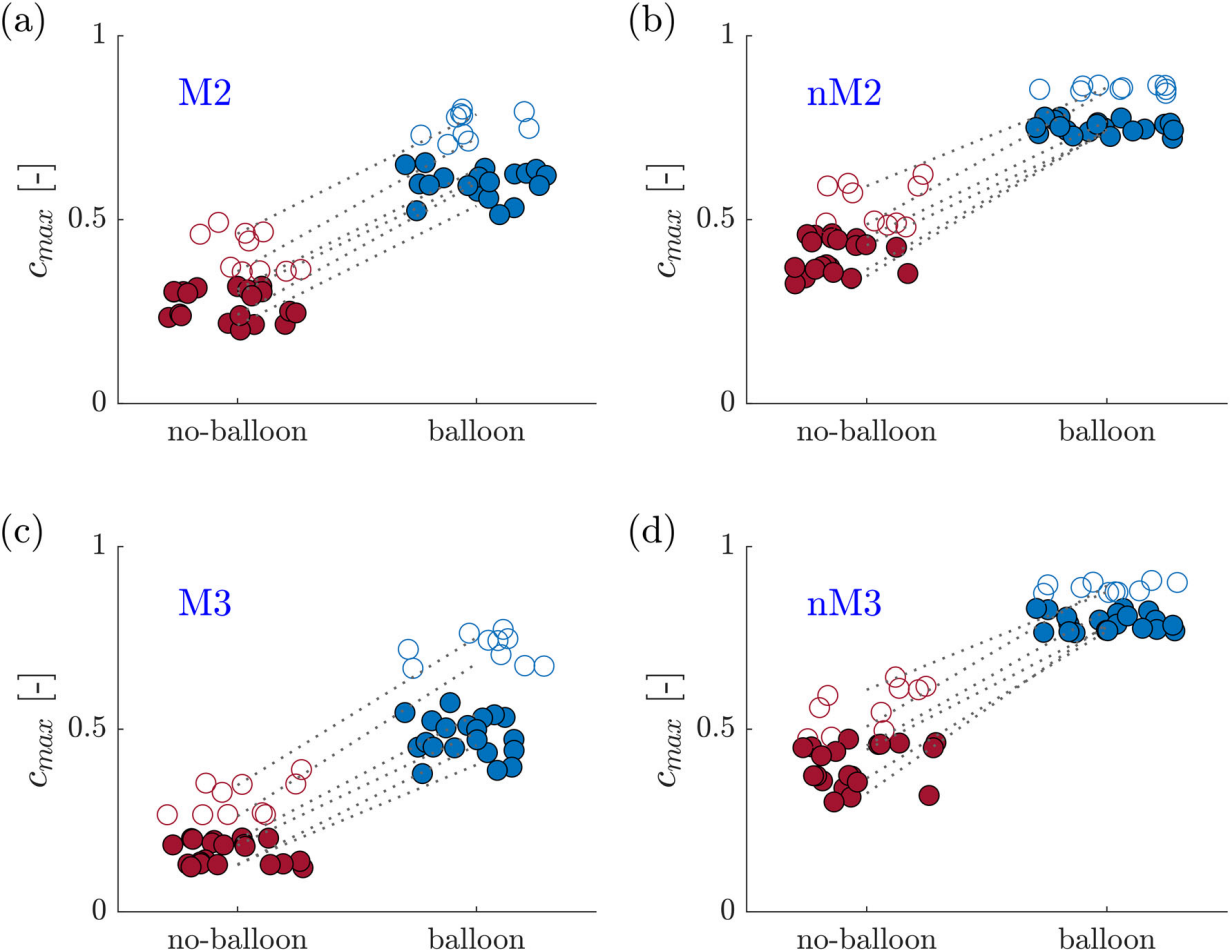
$c_{{{\text{max}}}}$ of the $50\, {\text{bpm}}$ (filled points) and the $69\, {\text{bpm}}$ (open circles) matching for the no-balloon (red) and the balloon (blue) case with a dotted line connecting the experimental day’s mean. (a) M2, (b) nM2, (c) M3, (d) nM3.

In Fig. 6, $c_{{{\text{max}}}}$ at *M2*, *M3*, *nM2*, *nM3* are shown for all experimental runs. The black dashed lines connect the means of $c_{{{\text{max}}}}$ on a given day (average over all five experiments performed on that day). Although there is a significant variation in $c_{{{\text{max}}}}$ over different experimental runs and different days of the experiment, the maximum concentrations for the balloon case are always higher than for the corresponding no-balloon case. Further, the following observations are made:

1. The $69\, {\text{bpm}}$ experiments (open circles) generally lead to higher $c_{{{\text{max}}}}$ than the $50\, {\text{bpm}}$ experiments (filled circles).
2. On the *no-MVO* side, concentrations at *nM2* are similar to concentrations at the more distal location *nM3* (for *no-balloon* and for *balloon*), whereas the more distal *M3* exhibits lower concentration values than *M2* on the *MVO* side.
3. In the *no-balloon* experiments, the concentrations on the *MVO* side are always lower than on the *no-MVO* side.
4. In the balloon experiments, the concentrations on the *MVO* side are higher than the concentrations on the *no-MVO* side in the *no-balloon* case.

**Figure 7:**
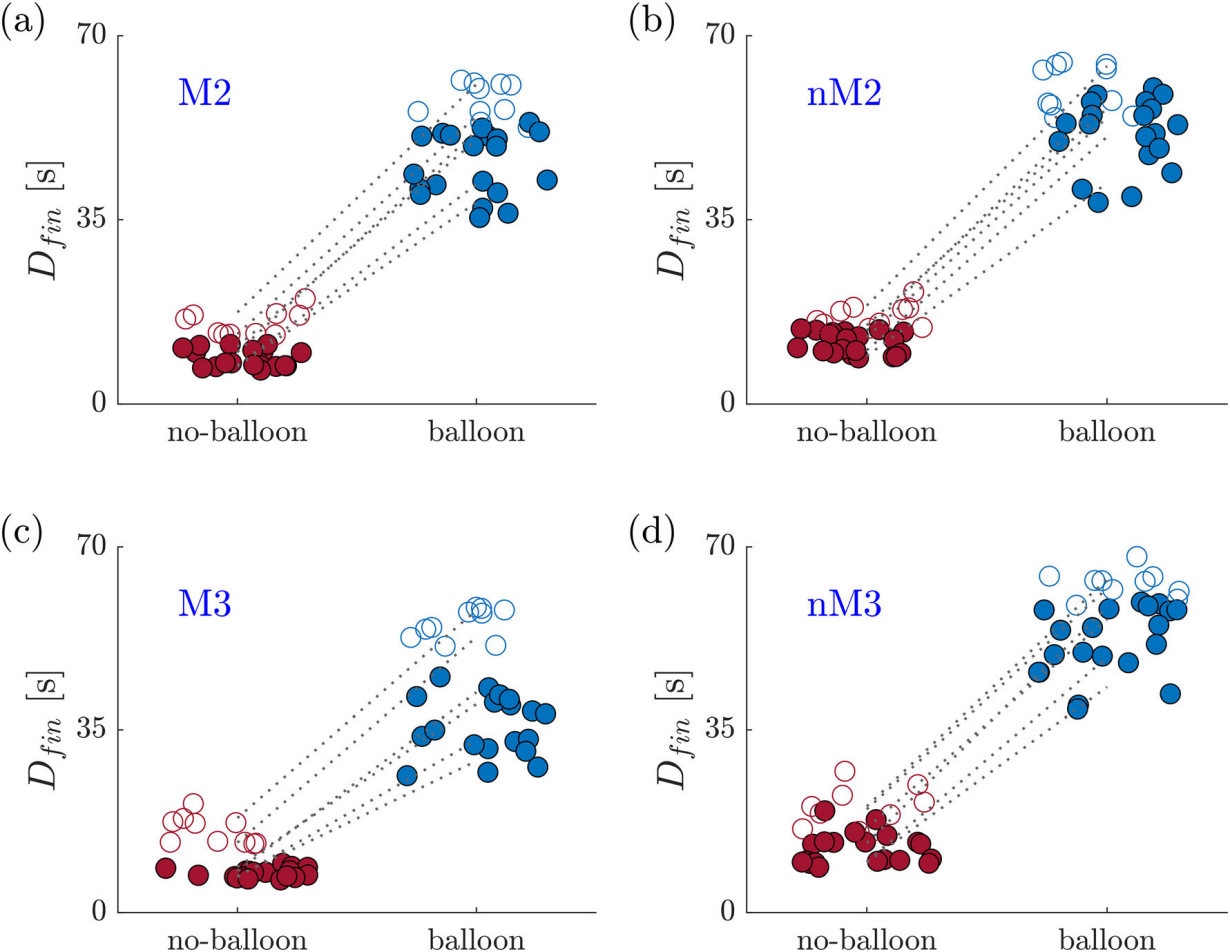
$D_{{{\text{fin}}}}$ for $50\, {\text{bpm}}$ (filled points) and $69\, {\text{bpm}}$ (open circles) for the no-balloon (red) and the balloon (blue) case. The dotted line connects the mean final doses per day.

These observations are also reflected the final cumulated doses $D_{{{\text{fin}}}}$ (Fig. 7).

**Figure 8:**
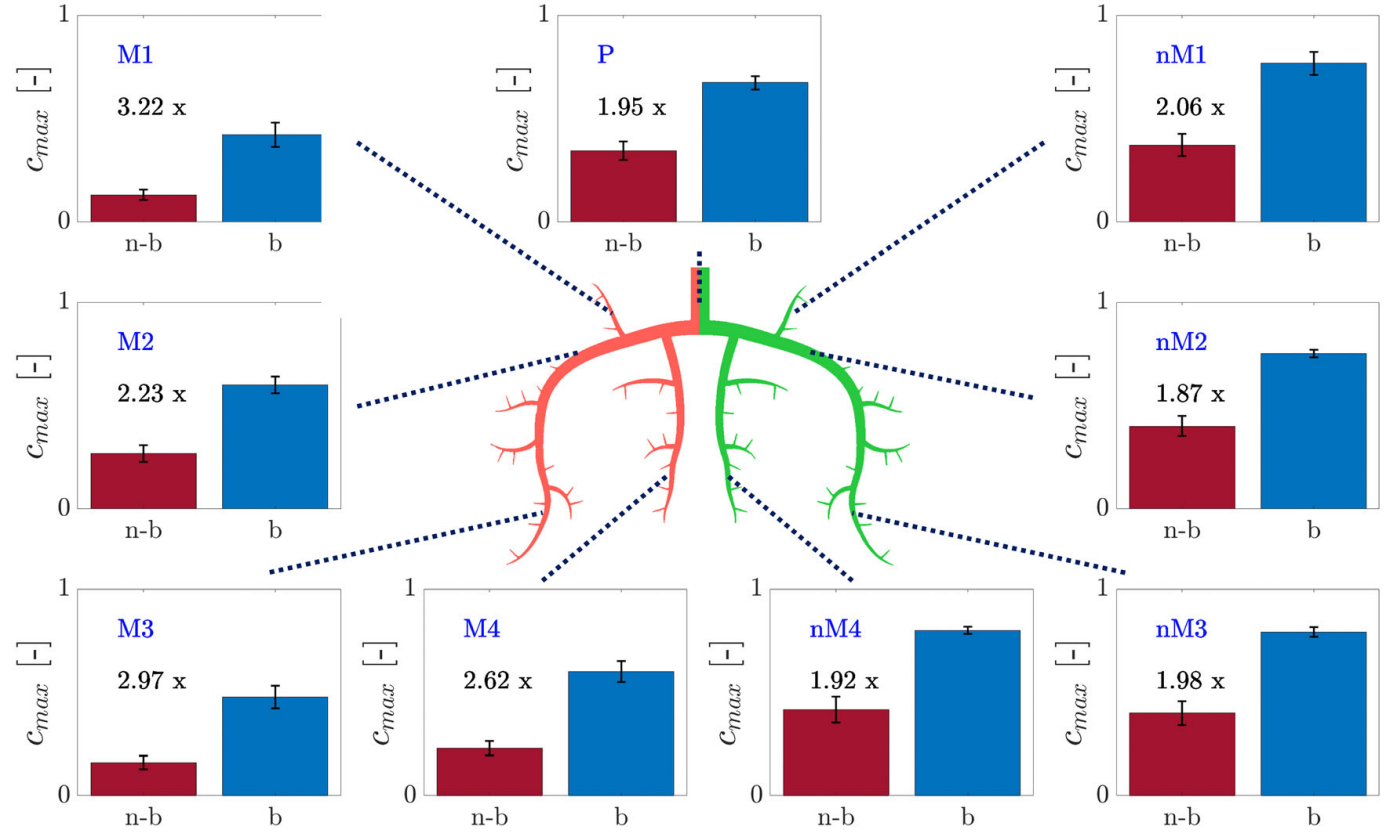
$c_{{\text{max,ba}}}$ and $c_{{\text{max,nb}}}$ with the corresponding factor by which $c_{{\text{max,ba}}}$ is higher than $c_{{\text{max,nb}}}$ at different sites in the microchip for the 50 bpm setting.

Figure 8 shows the mean $c_{{{\text{max}}}}$ (average over all experiments at $50\, {\text{bpm}}$) for the *balloon* and the *no-balloon* case for the locations *P*, *nM1*, …, *M4*. The factor indicates how much $c_{{{\text{max}}}}$ increased due to the balloon inflation. In general, this factor is higher on the *no-MVO* side and increases in smaller and more distal channels. Supplementary Figure S1 is the analog to Fig. 8 containing the corresponding factors for $D_{{{\text{fin}}}}$.

## Discussion

### Dye Transport

The results show that even if the resistors of the *MVO* side of the microchip completely block the outflow, there is a non-negligible amount of dye reaching these channels. This can be related to the action of the RC elements which yield an oscillatory volume displacement in the *MVO* channels due to myocardial contraction. Small amounts of fluid are pushed into proximal vessels during systole, mix with the local fluid of higher dye concentration, and are sucked in distal channels again during diastole. This process increases the drug concentration in the *MVO* channels with every heartbeat during the infusion phase.

For the *balloon* case, this transport mechanism into the *MVO* channels continues in the balloon-holding phase when the balloon is inflated but the infusion already stopped. The efficacy of this transport by pulsatile mixing depends strongly on the dye concentration at the proximal end of the blocked channels.

After the infusion stops, respectively when the balloon is deflated, the same transport mechanism goes in the other direction. The *no-MVO* side is washed out of dye quite fast, while the dye is washed out slowly on the *MVO* side.

The lower $c_{{{\text{max}}}}$ and $D_{{{\text{fin}}}}$ on the *MVO* side, plus the decreasing dye concentration in more distal areas point at the core of the problem of MVO treatment.

A dimensional analysis of the underlying flow phenomena in the model indicates that inertia and advection play significant roles in the observed drug transport despite the small scales of the studied vascular structures. Based on mass conservation and flow measurements in larger tubing of the coronary flow model, the mean flow velocities in the channels of the microchip could be estimated. These estimates yielded mean Reynolds numbers up to 18 in the largest vessel segment (order 9/parent vessel) and down to 4 in the small segments (order 5). Note that these values are representative of the vessels not affected by MVO. In the MVO-affected region, the flow velocities are smaller and so are the Reynolds numbers. Nevertheless, the shape of plumes of dye, that were issued from the smallest vessel segments into the pools (see Supplementary Figure S2), indicates that inertia remained dominant and that the local Reynolds numbers were (at least temporarily) above unity also in the smallest vessels affected by MVO. This is important because the reciprocal nature of the pulsating flow in the MVO-affected regions would make a net mass transport by mixing very difficult if the Reynolds numbers were smaller than unity. It is also important to note, that the Reynolds numbers would be three to four times smaller if the drugs were solved in blood because blood has a higher viscosity than water. Therefore, the use of a water-based drug solution is expected to be beneficial for the drug transport, because the higher Reynolds numbers support the mixing processes which are necessary to transport drug by reciprocal pulsatile flow.

The Womersley numbers associated with the vessel segments of the microchip are close to unity or below unity, such that the local flow field can be considered quasi-steady. Finally, the Peclet numbers in the chip are significantly larger than unity (assuming a diffusion coefficient on the order of $10^{ - 9} {\text{m}}^{{2}} {\text{/s}})$ which indicates that transport is dominated by advection rather than by diffusion.

### Influence of the Balloon Occlusion and Clinical Translation

Balloon inflation increases $c_{{{\text{max}}}}$ in the *MVO* channels by the factor of 2.2–3.2 (Fig. 8) and $D_{{{\text{fin}}}}$ increases even by the factor of 4.6–5.2 (Figure S1). This result is in line with Schwartz *et al*.^20^ who showed decreased vascular resistance after using the *balloon*-infusion protocol in an MVO animal model.

There are two factors leading to higher $c_{{{\text{max}}}}$ in the *MVO* region: first, due to the balloon occlusion, the treated vascular tree is temporarily disconnected from the bloodstream such that it takes longer until the drug is washed out. Drug transport in the blocked vessels is driven by the myocardial pump effect. The longer a high proximal concentration is present, the higher distal concentrations are possible. Second, the balloon occlusion not only prevents the drug from being washed out, but it also lowers the dilution of the drug with blood. By occluding the proximal vessel, and in absence of collateral vessels, the distal blood volume could theoretically be fully replaced by drug over time, because no new blood enters the treated vascular tree.

The increased concentrations for the $69\, {\text{bpm}}$ experiments (Fig. 7) could suggest that an increase in the heart rate may be beneficial for the drug delivery. A possible explanation for this effect may be based on the fact that a higher heart rate results in more volume displacement cycles which enhances mixing in the blocked vessels. However, this effect needs to be further investigated.

### Limitations

Figure 5 shows non-zero concentration values at the start of the experiments. These small variations (± 3%) are due to the uncertainty in the measurement of the concentration values from video frames and uncertainties in the associated calibration curves.

Figures 6 and 7 show a certain variability over the measured values for *c*_max_ and *D*_fin_. The variability remained small (5–7%, depending on the ROI) over one measurement day, but was significantly larger between different measurement days (13–27%, depending on the ROI). These differences may result from the calibration of the concentration measurement (e.g. small errors in the dilution of the dye on different measurement days) and from small changes in the settings of the impedance elements of the coronary model. This limits the reproducibility of the experiments and indicates that absolute concentration values should only be compared within measurements taken on the same day. At the same time, the relative concentration values, i.e. ratios between values from balloon and no-balloon experiments were reproducible also in experiments performed on different days.

Water was used as working fluid instead of blood (or a blood analogue fluid). In reality, the patient’s vessels are, at least in the beginning of the treatment, filled with blood that is approximately four times more viscous than water or a water-like drug solution. At the beginning of the infusion, it would be more difficult to bring drug into the small vessels filled with the more viscous blood. The longer the process is going on, the intramyocardial pump effect will reduce the effective viscosity by mixing the blood with water-like drug solution. To further assess the effect of blood viscosity, experiments with a blood analogue must be performed.

The performed infusion algorithm was simple and designed in a way to see the effects of the infusion phase and the balloon-holding phase separately. It is necessary to further study the influence of the infusion flow rate, the timepoint of balloon inflation as well as the duration of the balloon occlusion. It is possible that more efficient infusion algorithms can be identified. At the same time, the total balloon occlusion time should not be too long to not risk formation of new thrombi or oxygen-deficiency in the distal tissue.

The used microfluidic chip and MVO model had their limitations. The channels on the microfluidic chip were rigid and microvascular compliance was modelled by the distal RC elements. *Ad-hoc* tests without distal RC elements strongly reduced the distal compliance, such that there was almost no dye transport into the MVO-affected regions. Therefore, we believe that the RC elements are important to model the vascular compliance in the MVO-affected regions and that this distal compliance has a direct effect on the drug transport by mixing.

Finally, the MVO was introduced by distally blocking the channels instead of creating single blocked channels, mimicking local emboli. Therefore, our model represents only the situation slightly proximal to the actual emboli. This is still relevant because chances of drug reaching single occluded vessels will also rise by increased drug concentration in proximal parts of the microvascular network. Real MVO may be more complex (e.g. semi-permeable) and located at various levels within the microvascular tree or even blocking vascular bifurcations. Therefore, experiments with local obstructions in the chip (e.g. by small thrombi) should be performed.

## Conclusion

The results show that the application of an occlusion-infusion catheter for the intracoronary drug administration leads to a clear improvement compared to a simple intracoronary administration of the same amount of drug. It was possible to show that the maximal concentration $c_{{{\text{max}}}}$ as well as the final cumulated dose $D_{{{\text{fin}}}}$ increase by a factor of 2.2–3.2 and 4.6–5.2, respectively. The translation of these results to the catheter lab may result in more effective treatment of MVO.

### Electronic supplementary material

Below is the link to the electronic supplementary material.

Supplementary file1 Figure S1: D_fin,ba_ and D_fin,nb_ with the corresponding factor by which D_fin,ba_ is higher than D_fin,nb_ at different sites in the microchip for the 50 bpm setting (TIFF 32403 kb)

Supplementary file2 Figure S2: Unprocessed video frame of microchip during a balloon case experiment at 25 s. The detail view shows the plume shape of the dye in the pools on the MVO side. (TIFF 13672 kb)

Supplementary file3: Post-processed videos of a *no-balloon* and a *balloon* case. (MP4 138252 kb)

## Appendix

### Calibration

For the optical analysis of the dye concentration in the microchip, the experiment was calibrated on every experimental day. To this end, the microchip was mounted in the set-up and backlight illumination was adjusted so that the “white” areas of the empty microchip had RGB values around 240–250. The microchip was then uniformly filled with dye mixtures at concentrations of $0, 10, 20, 40, 60, 80, 100, 110 \%$ and then recorded with the camera. The data point at $110 \%$ was added to have one extra calibration point outside the used range. A concentration of 110% could be achieved, because the 100% solution was already a dilution of the used dye. All video frames were corrected for brightness (compared to the first frame), rotation and translation (compared to a master alignment frame).

Between the different concentrations the chip was flushed with water.

In the postprocessing of the video frame, a calibration curve was calculated for every pixel in the detected network structure (Fig. 4). To this end, the parameters *a*, *b*, *c* of the function

$$f\left( x \right) = { }\frac{1}{{a{*}\sqrt x + b}} + c$$

were optimized to minimize the root-mean-square error (RMSE),

$${\text{RMSE}} = \sqrt { \frac{1}{N} \mathop \sum \limits_{j = 1}^{N} \left[ {I_{j} - f\left( {x_{j} } \right)} \right]^{2} }$$

where $I_{j}$ was the mean pixel intensity (averaged over ten frames) at concentration $x_{j}$ and $N = 8$. Pixels for which no satisfactory calibration curve could be established were excluded from the analysis.
