# Supplementary figures and images for: Enhanced Drug Delivery for Cardiac Microvascular Obstruction with an Occlusion-Infusion-Catheter
Source: Ann Biomed Eng. 2023 Jan 21;51(6):1343–55. doi: 10.1007/s10439-023-03142-z (PMC10172228; doi:10.1007/s10439-023-03142-z)

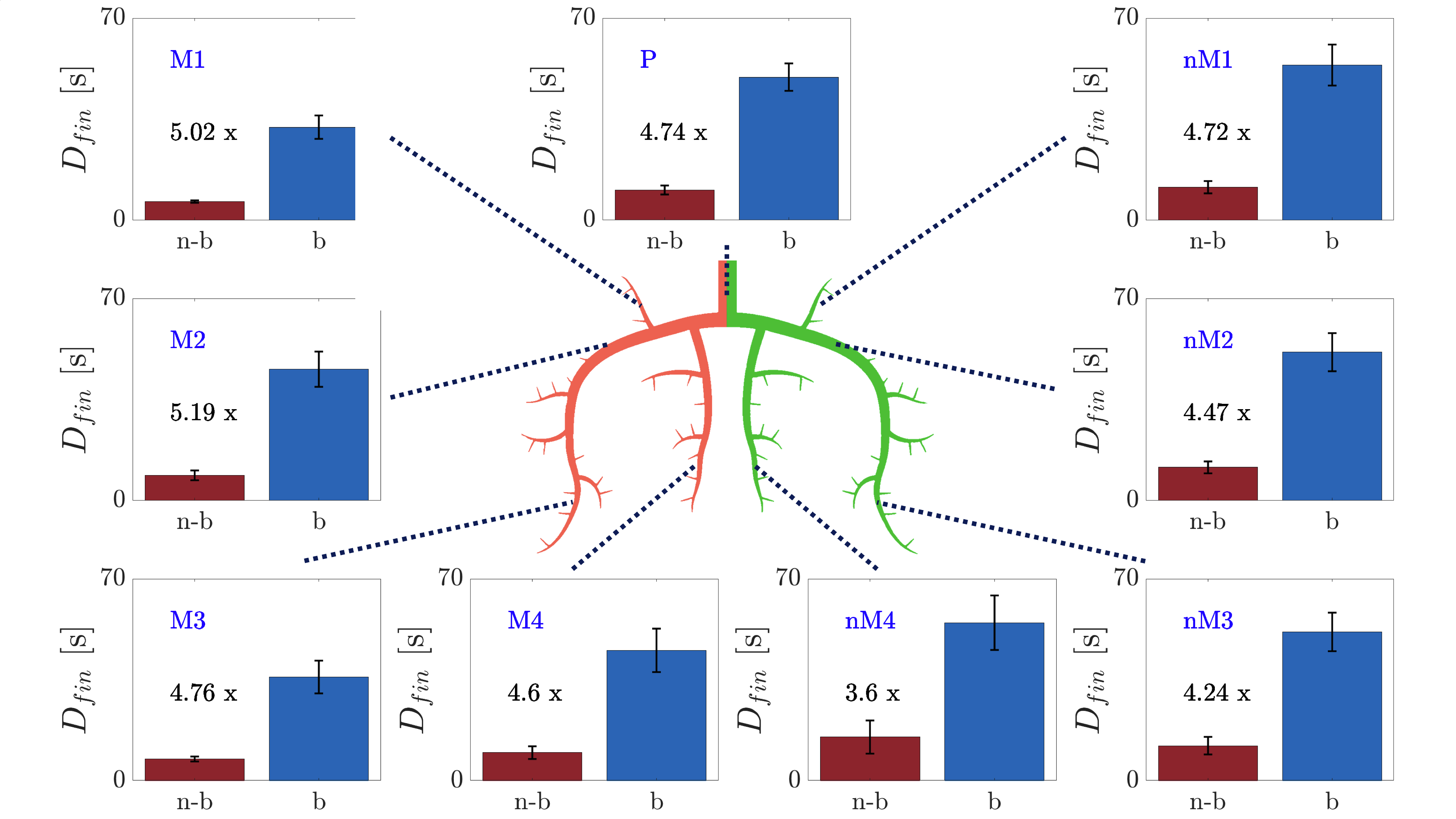

Supplement: Supplementary file 1 — Supplementary file1 Figure S1: Dfin,ba and Dfin,nb with the corresponding factor by which Dfin,ba is higher than Dfin,nb at different sites in the microchip for the 50 bpm setting (TIFF 32403 kb) [file 10439_2023_3142_MOESM1_ESM.tiff]

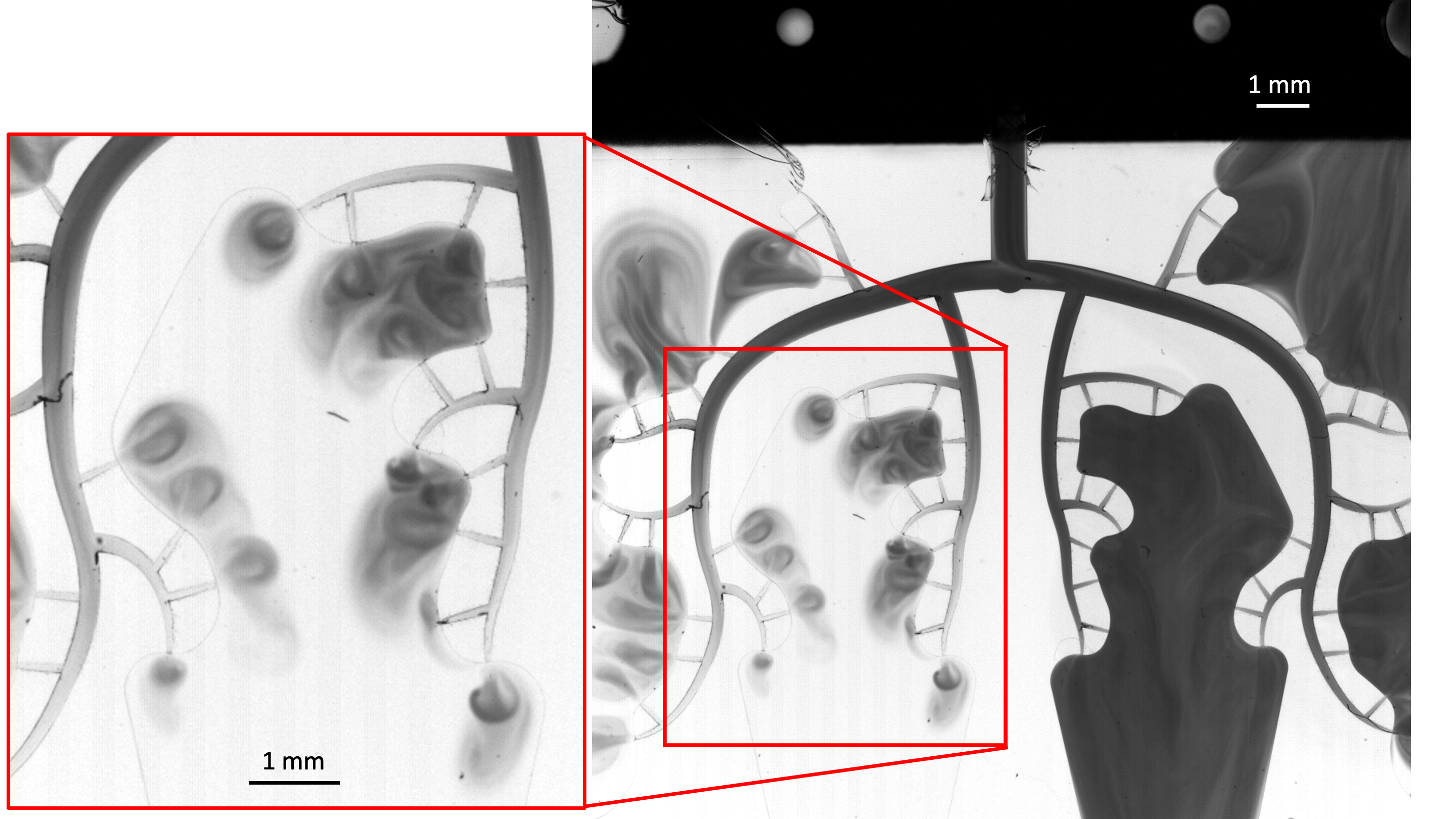

Supplement: Supplementary file 2 — Supplementary file2 Figure S2: Unprocessed video frame of microchip during a balloon case experiment at 25 s. The detail view shows the plume shape of the dye in the pools on the MVO side. (TIFF 13672 kb) [file 10439_2023_3142_MOESM2_ESM.tiff]
